# Gestational Diabetes Mellitus: Predictive Value of Fetal Growth Measurements by Ultrasonography at 22–24 Weeks: A Retrospective Cohort Study of Medical Records

**DOI:** 10.3390/nu12123645

**Published:** 2020-11-27

**Authors:** Danyao Jin, Janet Wilson Rich-Edwards, Chunyi Chen, Yue Huang, Yinping Wang, Xiangrong Xu, Jue Liu, Zheng Liu, Yaqing Gao, Siyu Zou, Hong Zhou, Haijun Wang

**Affiliations:** 1 Department of Maternal and Child Health, School of Public Health/National Health Commission Key Laboratory of Reproductive Health, Peking University, Beijing 100191, China; danyao_jin@hsph.harvard.edu (D.J.); chenchunyi199558@126.com (C.C.); huangyue0103@163.com (Y.H.); wyping201406@bjmu.edu.cn (Y.W.); xuxiangrong@bjmu.edu.cn (X.X.); jueliu@bjmu.edu.cn (J.L.); liuzheng@bjmu.edu.cn (Z.L.); gaoyaqing@bjmu.edu.cn (Y.G.); zousiyu@pku.edu.cn (S.Z.); whjun1@bjmu.edu.cn (H.W.); 2 Department of Epidemiology, Harvard T.H. Chan School of Public Health, Boston, MA 02115, USA; jr33@partners.org; 3 Division of Women’s Health, Brigham and Women’s Hospital and Harvard Medical School, Boston, MA 02115, USA

**Keywords:** fetal growth, fetal ultrasound, gestational diabetes mellitus, prenatal screening, pregnancy nutrition

## Abstract

Early intervention of gestational diabetes mellitus (GDM) is effective in reducing pregnancy disorders. Fetal growth, measured by routine ultrasound scan a few weeks earlier before GDM diagnosis, might be useful to identify women at high risk of GDM. In the study, generalized estimating equations were applied to examine the associations between ultrasonic indicators of abnormal fetal growth at 22–24 weeks and the risk of subsequent GDM diagnosis. Of 44,179 deliveries, 8324 (18.8%) were diagnosed with GDM between 24 and 28 weeks. At 22–24 weeks, fetal head circumference (HC) < 10th, fetal femur length (FL) < 10th, and estimated fetal weight (EFW) < 10th percentile were associated with 13% to 17% increased risks of maternal GDM diagnosis. Small fetal size appeared to be especially predictive of GDM among women who were parous. Fetal growth in the highest decile of abdominal circumference (AC), HC, FL and EFW was not associated with risk of subsequent GDM. The observed mean difference in fetal size across gestation by GDM was small; there was less than 1 mm difference for AC, HC, and FL, and less than 5 g for EFW before 24 weeks. Despite similar mean fetal growth among women who were and were not later diagnosed with GDM, mothers with fetuses in the lowest decile of HC, FL and EFW at 22–24 weeks tended to have higher risk of GDM.

## 1. Introduction

Gestational diabetes mellitus (GDM) is a carbohydrate intolerance resulting in hyperglycaemia of variable severity with onset or first recognition during pregnancy [1]. GDM affects 10–26% of pregnancies throughout the world [2]. The prevalence of GDM is high in Chinese women, estimated at 14.8% in mainland China according to a meta-analysis of 25 papers between 2010 and 2017 [3]. GDM is associated with adverse health outcomes for both mothers and their offspring, including preeclampsia, preterm birth and macrosomia [4,5,6]. In the long term, women with a history of GDM are at increased risk of type 2 diabetes and cardiovascular disease [7,8]. In addition, offspring born to mothers with GDM are prone to childhood and adolescent obesity and metabolic dysregulation [9,10,11].

Evidence suggests that the early detection and intervention of GDM is effective in reducing macrosomia, pre-eclampsia and hypertensive disorders in pregnancy [1,12,13]. Current guidelines recommend screening women for GDM between 24 and 28 weeks of gestational age (wkGA), with earlier screening for high-risk women (marked obesity, personal history of GDM, glycosuria, or a strong family history of diabetes) [1,2,14,15]. Most pregnant women have their second-time routine perinatal ultrasound scan before 24 wkGA; if fetal growth is able to identify women at high risk of GDM, it might be useful to identify women who should be screened early. To date, one study has examined fetal growth as a predictor of GDM onset, finding no association of estimated fetal weight at 18–22 weeks with subsequent GDM diagnosis [16]. Other studies focused on GDM as a predictor of fetal growth have included some fetal growth measurements likely taken before GDM diagnosis. In these studies, GDM diagnosed after 24 wkGA had inconsistent associations (positive [17], inverse [18,19], or null [20]) with fetal growth measures taken before 24 wkGA. There is some evidence that the effect of GDM on fetal growth is increased with maternal obesity [20]; but no study has yet explored the interaction of GDM with other factors, for example, parity and maternal age. Therefore, we aimed to determine whether abnormal fetal growth at 22–24 weeks among the Chinese population predicts GDM onset and whether that risk varied by maternal age, pre-pregnancy obesity and parity.

## 2. Methods

### 2.1. Design and Participants

Maternal demographic, anthropometric and fetal sonographic data were collected from the hospital medical record system for all women attending antenatal care and delivery in Tongzhou Maternal and Child Health Hospital, from January 2012 to December 2018. Eligible women had: (1) a viable singleton pregnancy, (2) fetal sonographic data during 22–24 wkGA, and (3) a glucose tolerance test between 24–28 wkGA. Women whose ultrasound scan and glucose test were performed in the same week (*n* = 279) or those missing fetal sonographic measurements (*n* = 42) were excluded. Maternal height and pre-pregnancy weight were imputed for 15,455 deliveries. Ethics approval was given by the Medical Research Ethics Committee of Peking University (Certificate references: IRB00001052-18004).

### 2.2. Data Collection and Definitions

Maternal age, parity and fetal sex were obtained from the hospital information system. Self-reported pre-pregnancy weight and clinically measured maternal height were recorded during the first perinatal visit. Pre-pregnancy body mass index (BMI) was calculated as pre-pregnancy weight in kilograms divided by height in meters squared. Gestational age was calculated based on self-reported last menstrual period and confirmed by first-trimester ultrasonography.

Fetal Biometry: Fetal head circumference (HC, cm), abdominal circumference (AC, cm), and femur length (FL, cm) were measured by ultrasound scan at 22–24 wkGA. Estimated fetal weight (EFW, g) was calculated by Hadlock formula using HC, AC, and FL (the formula used by WHO fetal biometry reference) [21,22]. The percentiles of HC, AC, FL and EFW (gestational age specific) were defined by WHO fetal biometry reference [22]. The highest and lowest deciles of HC, AC, FL and EFW at each time point were defined as overgrowth and undergrowth.

Diagnosis of GDM: a 75-g oral glucose tolerance test (OGTT) at 24–28 wkGA was used to diagnose GDM. Based on diagnostic criteria adapted from the International Association of Diabetes and Pregnancy Study Groups (IADPSG) in 2010 [23], fasting plasma glucose (FPG) level ≥ 5.1 mmoL/L or 1-h plasma glucose (1hPG) level ≥ 10.0 mmoL/L or 2-h plasma glucose (2hPG) level ≥ 8.5 mmoL/L defined GDM diagnosis.

### 2.3. Statistical Analysis

Differences in demographics and proportion of abnormal fetal growth between women with and without GDM were compared using t-tests for continuous variables and Chi-square tests for categorical variables. Generalized estimating equations (GEE) were applied to examine the associations between indicators of abnormal fetal growth and GDM; this allowed us to account for correlations between pregnancies for the 3756 women with more than one pregnancy during 2012-2018. The indicators of fetal overgrowth or undergrowth for each fetal anthropometric measurement were entered respectively as exposures. Maternal age, parity, fetal sex, maternal height and pre-pregnancy BMI were then included to obtain adjusted odds ratios and 95% confidence intervals. Missing values for maternal height and pre-pregnancy BMI were imputed using multiple imputation. As a sensitivity analysis, we reran the above models using the INTERGROWTH-21st standard and a Chinese standard to define extremes of fetal growth [24,25]. To compare the mean difference in fetal growth across gestation by GDM, the observed fetal growth trajectories were plotted and t-tests comparing mean fetal growth at each timepoint by final GDM status were performed. Stratified ORs were calculated by: pre-pregnancy BMI (<25 kg/m^2^ vs. ≥25 kg/m^2^); parity (nulliparous vs. parous); and age (<30 vs. ≥30). All analyses were conducted using SAS, version 9.4 (SAS Institute). Statistical significance was considered at a two-tailed *p*-value of < 0.05.

## 3. Results

Of 44,179 deliveries, 8324 (18.8%) were diagnosed with GDM before 28 wkGA. Women with GDM were significantly older, taller and heavier, more likely to be parous, and bore offspring of heavier birth weight (Table 1).

The mean weeks gestation for ultrasound scan and glucose test were 23.2 ± 0.5 weeks and 26.3 ± 0.8 weeks, respectively. In crude analysis, mothers with fetuses in the lowest decile of fetal HC and FL at 22–24 wkGA had statistically significant 11% and 14% increased risk of subsequent GDM compared to mothers with normal fetal growth at 22–24 wkGA. The risk of developing GDM did not vary by fetal growth parameters among mothers with fetuses in the highest decile (Table 2). After adjusting for fetal sex, maternal age and parity, those in the lowest decile of fetal HC, FL and EFW had higher risks of subsequent GDM. After additional adjustment for maternal height and pre-pregnancy BMI, women whose fetuses were in the lowest decile of HC, FL and EFW had odds ratios of 1.15 [95%CI 1.06,1.25], 1.17 [95%CI 1.08,1.27], and 1.13 [95%CI 1.03,1.25], respectively, of being diagnosed with subsequent GDM. Fetal growth parameters in the highest decile were not associated with the risk of subsequent GDM (Table 2). The values of adjusted odds ratios for most measures were consistent with the results based on INTERGROWTH-21st standard and Chinese standard. The only exception was the lowest decile of EFW based on INTERGROWTH-21st standard, with odds ratio of 1.05 [95%CI 1.00,1.11], which is lower than the results using WHO standard (1.13 [95%CI 1.03,1.25]) and Chinese standard (1.14 [95%CI 0.99,1.31]) (Supplementary Tables S2 and S3).

Although the interaction terms of GDM and parity did not reach statistical significance (Table 3), ORs for fetal undergrowth appeared stronger for parous women, who had 28%, 34% and 26% higher odds of GDM if they carried fetuses < 10th percentile of HC, FL, and EFW, respectively. With the exception of a 12% increased odds of GDM associated with FL < 10th percentile, these associations were not evident for nulliparous mothers. There was no evidence of effect measure modification for obesity and maternal age to the risk of GDM associated with low fetal growth.

**Figure 1:**
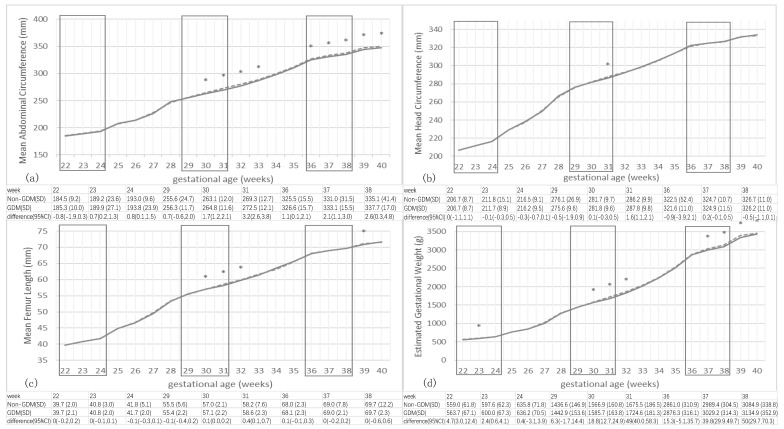
Comparison of mean fetal biometry in gestational diabetes mellitus (GDM) and non-GDM mothers by gestational age. Mean Head circumference (**a**), biparietal diameter (**b**), abdominal circumference (**c**) and femur length (**d**) of fetuses in pregnancies ultimately diagnosed with GDM (dashed line) vs. pregnancies that remained normoglycemic (solid line), by gestational age. * Significant difference (*p* < 0.05) in size between GDM-exposed fetuses and unexposed fetuses. Three squares in each plot indicate weeks in which routine ultrasound scans were typically performed.

Figure 1 shows the mean fetal size across gestation by final GDM status. Before 30 wkGA, mean AC, HC and FL were indistinguishable between pregnancies with and without GDM; mean differences were <1 mm and not statistically significant. After this point, fetuses from GDM pregnancies tended to have slightly larger AC than fetuses from non-GDM pregnancies, and achieved the largest mean difference of 3.2 [95%CI 2.6, 3.8] mm at 31 weeks. Similarly, mean EFW for women with and without GDM did not diverge until 30 wkGA; the one distinction was a slightly higher mean EFW at 23 weeks (2.4 [95%CI 0.2, 4.2] g difference) among those later diagnosed with GDM. As with AC, after 30 weeks, fetuses exposed to GDM were slightly heavier than those without GDM.

## 4. Discussion

### 4.1. Key Results

In this study we observed that the ultrasound-measured growth of Chinese fetuses at 22–24 wkGA was associated with GDM onset in their mothers a mean of 3 weeks before the biochemical diagnosis of GDM. In particular, the lowest decile of head circumference, femur length and estimated fetal weight were associated with 13% to 17% higher risk of subsequent GDM after adjusting for fetal sex, maternal age, parity, pre-pregnancy BMI and maternal height. The association with small HC, FL and EFW was especially evident among parous mothers, with 28%, 34% and 26% higher risks, respectively. These differences in the extremes of the fetal growth distributions were not reflected in differences in mean fetal biometry measures, and might have been missed if the focus had been on population averages.

### 4.2. Interpretation

Only one study, based on a Canadian population, has examined estimated fetal weight as a predictor of later GDM diagnosis, showing no association [18]. While other studies have included data on GDM and fetal growth, their focus has been on predicting fetal growth or comparing the difference in fetal growth trajectories of women after GDM testing, not on predicting GDM from early fetal growth [17,18,19,20]. However, such studies may shed some light on the associations we observed. One British study suggested no association between fetal biometry measured at 20 wkGA and later GDM diagnosis; this study did, however, observe excessive growth of AC between 20 and 28 wkGA for fetuses of women diagnosed with GDM [20]. In contrast, another study reported that pregnancies later diagnosed with GDM had statistically significant 1 mm greater mean AC at 16–18 weeks among Black South African women [17]. Two studies in mixed European and Asian populations appear to be consistent with our study, reporting decreased fetal growth at 12–24 weeks among women later diagnosed with GDM: one reported less than 0.5 standard deviations smaller fetal size associated with GDM, and the other reported less than 2 mm difference in mean AC and HC, and less than 10 g for mean EFW in women later diagnosed with GDM [18,19]. Thus, there is limited and conflicting evidence across populations regarding the direction and magnitude of differences in fetal size of pregnancies subsequently diagnosed with GDM. The seeming conflict may reflect differences in race/ethnicity in associations between early fetal growth and subsequent GDM diagnosis. Future studies examining fetal growth as a predictor of GDM should be conducted in various ethnic populations.

At 22–24 wkGA, before GDM diagnosis, we observed no differences in mean fetal size between those who would later be diagnosed with GDM and those who remained normoglycemic; however, by 30 wks GA, the fetuses of women diagnosed with GDM in the interval had grown larger; this suggests that fetal growth accelerates more rapidly in GDM pregnancies around the time of diagnosis. This more rapid acceleration in fetal growth from the period before GDM diagnosis to the period after GDM diagnosis is consistent with three of the studies with data on fetal growth before and after diagnosis [18,19,20].

Despite contradictory evidence regarding the association of fetal growth with later GDM diagnosis, studies are nearly unanimous that fetal growth after GDM diagnosis is accelerated. This poses the question whether the drivers of fetal growth are the same before and after GDM diagnosis, as suggested by a study among women with GDM and impaired glucose tolerance, which found that predictors of fetal growth varied between 24 and 36 wkGA [26]. One possible mechanism linking reduced early fetal growth with later increased risk of GDM might be placental growth hormone-variant (GH-V). GH-V mobilizes maternal nutrients for fetal growth by inducing maternal insulin resistance. GH-V deficiency may impede the mobilization of maternal nutrient stores and reduce maternal insulin-like growth factor 1 (IGF-1) concentrations, thereby limiting uterine growth, placental blood flow, and transplacental nutrient transfer and in turn limiting fetal growth [27]. Glucose has been recognized as the regulator of placental GH-V secretion [28,29] and high glucose levels are reported to decrease GH-V secretion [28]. Mothers with GDM may have high glucose levels before 24 weeks that may affect fetal growth before GDM diagnosis. However, to marshal this explanation for reduced fetal growth before GDM requires that the mechanism reverse after GDM diagnosis, when high maternal blood glucose predicts accelerated fetal growth and macrosomia. This possibility is suggested by a study of pregnancies complicated by GDM and impaired glucose tolerance, which found that FPG predicted fetal AC only after 32 wkGA; at 24 and 28 wksGA, only maternal BMI and history of previous LGA births predicted fetal growth [26]. At present, the mechanisms linking restricted fetal growth with increased risk of GDM are speculative and need further exploration.

We found a suggestion that fetal undergrowth was associated with increased risk of GDM especially in women who were parous, independent of maternal age and prepregnant BMI. Every additional pregnancy can diminish the β-cell reserve, generally contributing to maternal glucose intolerance and insulin resistance which promote fetal growth [30,31]. It is possible that fetal growth restriction in a parous population, where it is less common, may indicate the presence of comorbidities, such as preeclampsia, that are associated both with reduced fetal growth and increased risk of GDM.

Our data suggest that abnormal fetal growth happening during routine fetal biometry before the biochemical diagnosis of GDM might indicate a higher risk of subsequent GDM onset. We observed fetal growth accelerates rapidly around the time of GDM diagnosis; if replicated, fetal growth measures might be used to identify pregnancies that would benefit from earlier GDM screening and intervention. However, this association of fetal abnormal growth and GDM diagnosis needs to be replicated in future studies. It remains to be seen whether a 10% to 20% increased risk of GDM associated with the 10th percentile threshold of fetal undergrowth could prove clinically useful as a screening tool to identify women who would benefit from earlier GDM testing; its impact on net reclassification improvement or the area under receiver operating characteristic (ROC) curve are likely to be modest. Furthermore, the advantages of advancing GDM diagnosis by a few weeks are as yet unproven.

Besides WHO standard, we reran the model using the INTERGROWTH-21st standard and the Chinese standard, and received consistent odds ratios for all three standards among most of biometry measures. The only exception was EFW in the lowest decile based on INTERGROWTH-21st standard, which showed less increased risk of GDM compared to the results based on two other standards. It is worth noting that the 10th and 90th percentile of each standard identifies a different population as having abnormal fetal growth, and that difference was most obvious for EFW measures. For example, as evident in Table S1, roughly 33% of fetuses had EFW < 10th percentile by the INTERGROWTH-21st standard, compared to 7% by the WHO standard and 3% by Chinses standard. Furthermore, since the updated version of WHO reference was only recently published (in 2017), most previous studies examining the association of fetal growth and GDM utilized the INTERGROWTH-21st standard [17,18,19,20]. The INTERGROWTH-21st standard includes the Chinese population as one of eight study populations; however, previous authors have suggested that the INTERGROWTH-21st standard misclassifies a significant number of fetuses with fetal growth restriction, especially for the head circumference standard [32,33]. One possible reason for this discrepancy is that the INTERGROWTH-21^st^ project excluded women with BMI less than 18.5 kg/m^2^ [22]; more than 7% of pregnant women in our study would have been ineligible for the INTERGROWTH-21st project. The updated WHO standard, based on a wider range of study population and broadened the inclusion criteria to BMI ≥ 18 kg/m^2^, would have excluded only 3% of our population.

### 4.3. Strength and Limitations

The study has several strengths. To our knowledge, this is the largest study of fetal growth and GDM in an Asian population, which has a particularly high incidence of GDM. This is also the first study to examine an interaction of parity and fetal growth on the risk of GDM onset. Furthermore, the adoption of an updated version of the WHO fetal biometry reference allows for more accurate measurement than previous studies. The study also has several limitations. Pre-existing diabetes was not excluded from the study population because this information was unavailable from the database. However, considering a prevalence of 5.9% of chronic diabetes among the Chinese population less than 40 years old [34], the likelihood that chronic diabetes drove the association we observed between fetal growth restriction and GDM seems small. Finally, since the study included participants from only one hospital in Beijing, the results may not be generalizable to the whole Chinese population.

## 5. Conclusions

In summary, this study demonstrated a statistically significant association between fetal undergrowth at 22–24 wkGA and subsequent GDM diagnosis in China. Mothers with fetal HC, FL and EFW in the lowest decile tended to have higher risk of GDM after adjusting for fetal sex, maternal age, parity, pre-pregnancy BMI and maternal height. Future studies should examine whether this increased risk is clinically useful to identify women for earlier GDM screening. More clinical utility may be derived from examining extremes of fetal growth than mean population differences.

## Figures and Tables

**Table 1:** Characteristics of the study population by GDM.

Factor	No GDM (*n* = 35,855)	GDM (*n* = 8324)	*p* *
Age, mean years (SD)	28.6 (3.9)	29.7 (4.2)	<0.001
Age Categories, *n* (%)			<0.001
<25	5129 (14)	729 (9)	
25 to <30	17,495 (49)	3567 (43)	
30 to <35	10,482 (29)	2851 (34)	
≥35	2749 (8)	1177 (14)	
Parous, *n* (%)	10,777 (30)	2778 (33)	<0.001
Maternal Height, mean cm (SD)	162.0 (4.6)	161.6 (4.7)	<0.001
Maternal Height Categories, *n* (%)			<0.001
<160	5503 (24)	1469 (27)	
160 to <165	10,614 (46)	2444 (44)	
165 to <170	5631 (24)	1258 (23)	
≥170	1577 (7)	336 (6)	
Missing	12,530 (35)	2817 (34)	
Pre-pregnancy BMI kg/m^2^, mean (SD)	21.8 (3.1)	23.0 (3.3)	<0.001
Pre-pregnancy BMI Categories, *n* (%)			<0.001
Underweight (<18.5)	2894 (8)	352 (4)	
Normal (18.5 to <25)	17,141 (48)	3768 (45)	
Overweight (25 to <30)	2841 (8)	1168 (14)	
Obese (≥30)	369 (1)	191 (2)	
Missing	12,610 (35)	2845 (34)	
Gestational Length, mean weeks (SD)	40.1 (1.4)	39.8 (1.5)	<0.001
Male Fetus, *n* (%)	18,503 (52)	4337 (52)	0.41
Birth Length, mean cm (SD)	50.1 (1.4)	50.0 (1.6)	0.28
Birth Weight, mean g (SD)	3384 (445)	3418 (485)	<0.001
Birth Weight Categories, *n* (%)			<0.001
<2500	869 (3.5)	247 (4.2)	
2500 to <4000	20,916 (84.9)	4771 (81.0)	
≥4000	2848 (11.6)	872 (14.8)	
Missing	11,222 (31)	2434 (30)	

* continuous data are presented as mean (SD) and compared using Student t test; categorical variables are presented as frequency, *n* (%), and compared using x^2^ tests.

**Table 2:** Association between fetal biometry at 22–24 gestational weeks and subsequent gestational diabetes (GDM).

Main Exposure	GDM, *n* (%)	Crude OR (95% CI)	Adjusted OR ^†^ (95% CI)	Adjusted OR ^‡^ (95% CI)
AC				
<10th percentile (*n* = 2442)	436 (17.9)	0.94(0.84, 1.04)	1.00 (0.90, 1.12)	1.00 (0.89, 1.11)
10th–90th percentile (*n* = 36,036)	6773 (18.8)	1.0 (reference)		
>90th percentile (*n* = 5701)	1115 (19.6)	1.05 (0.98, 1.12)	0.99 (0.92, 1.06)	0.98 (0.91, 1.05)
HC				
<10th percentile (*n* = 4219)	856 (20.3)	1.11 (1.02, 1.20) *	1.17 (1.08, 1.27) *	1.15 (1.06, 1.25) *
10th–90th percentile (*n* = 35,299)	6599 (18.7)	1.0 (reference)		
>90th percentile (*n* = 4661)	869 (18.6)	1.00 (0.92, 1.08)	0.95 (0.88, 1.03)	0.97 (0.90, 1.05)
FL				
<10th percentile (*n* = 4069)	842 (20.7)	1.14 (1.05, 1.23) *	1.21 (1.12, 1.31) *	1.17 (1.08, 1.27) *
10th–90th percentile (*n* = 37,350)	6948 (18.6)	1.0 (reference)		
>90th percentile (*n* = 2760)	534 (19.3)	1.05 (0.95, 1.16)	1.02 (0.92, 1.12)	1.01 (0.91, 1.11)
EFW				
<10th percentile (*n* = 3010)	599 (19.9)	1.09 (0.99, 1.19)	1.16 (1.05, 1.27) *	1.13 (1.03, 1.25) *
10th–90th percentile (*n* = 33,614)	6245 (18.6)	1.0 (reference)		
>90th percentile (*n* = 7555)	1480 (19.6)	1.07 (1.00, 1.14) *	1.01 (0.94, 1.07)	1.00 (0.94, 1.07)

* *p* < 0.05 † Adjusted for maternal age, parity and fetal sex. ‡ Adjusted for maternal age, parity, fetal sex, pre-pregnancy height and pre-pregnancy BMI. OR = odds ratio; AC = abdominal circumference; HC = head circumference; FL = femur length; EFW = estimated fetal weight.

**Table 3:** Stratified odds ratios (OR) of GDM by parity and test of interactions between fetal biometry at 22–24 weeks gestation and parity.

Fetal Growth at 22–24 Weeks Gestation	Adjusted OR †		*p*-Value of Interaction Term
	Nulliparous	Parous	
AC < 10th percentile	0.99 (0.87, 1.13)	1.03 (0.85, 1.26)	0.74
HC < 10th percentile	1.10 (1.00, 1.21)	1.28 (1.10, 1.48) *	0.11
FL < 10th percentile	1.12 (1.02, 1.24) *	1.34 (1.15, 1.55) *	0.08
EFW < 10th percentile	1.09 (0.97, 1.22)	1.26 (1.06, 1.49) *	0.20
AC > 90th percentile	0.99 (0.91, 1.09)	0.96 (0.85, 1.09)	0.71
HC > 90th percentile	0.96 (0.87, 1.07)	0.98 (0.86, 1.13)	0.85
FL > 90th percentile	1.09 (0.97, 1.23)	0.87 (0.73, 1.03)	0.04 *
EFW > 90th percentile	1.03 (0.95, 1.12)	0.95 (0.85, 1.06)	0.27

* *p* < 0.05, † Adjusted for maternal age, fetal sex, pre-pregnancy height and pre-pregnancy BMI. OR = odds ratio; AC = abdominal circumference; HC = head circumference; FL = femur length; EFW = estimated fetal weight.

## Supplementary Materials

The following are available online at https://www.mdpi.com/2072-6643/12/12/3645/s1, Table S1 Proportions of fetuses in this Chinese population that would be classified with extreme fetal growth by the WHO standard, INTERGROWTH-21st standard, and Chinese standard; Table S2 Association between fetal biometry at 22–24 gestational week and subsequent GDM based on INTERGROWTH-21st standard; Table S3 Association between fetal biometry at 22–24 gestational weeks and subsequent GDM based on Chinese standard
