# Supplementary material for: Gestational Diabetes Mellitus: Predictive Value of Fetal Growth Measurements by Ultrasonography at 22–24 Weeks: A Retrospective Cohort Study of Medical Records
Source: Nutrients. 2020 Nov 27;12(12):3645. doi: 10.3390/nu12123645 (PMC7760346; doi:10.3390/nu12123645)
Supplement: Supplementary file 1 [file nutrients-12-03645-s001.pdf]

Table S1. Proportions of fetuses in this Chinese population that would be classified with extreme fetal growth by the WHO standard, INTERGROWTH -21st standard, and Chinese standard.

	WHO standard			INTERGROWTH-21st standard			Chinese standard		
Factor	Non-GDM, n (%)	GDM, n (%)	p*	Non-GDM, n (%)	GDM, n (%)	p*	Non-GDM, n (%)	GDM, n (%)	p*
AC			0.17			0.16			0.17
<10th percentile	2,006 (5.6)	436 (5.2)		1,921 (5.4)	408 (4.9)		2,740 (7.6)	594 (7.1)	
10th-90th percentile	29,263 (81.6)	6,773 (81.4)		28,177 (78.6)	6,538 (78.5)		30,437 (84.9)	7,078 (85.0)	
>90th percentile	4,586 (12.8)	1,115 (13.4)		5,757 (16.1)	1,378 (16.6)		2,678 (7.5)	652 (7.8)	
HC			0.041			0.021			0.087
<10th percentile	3,363 (9.4)	856 (10.3)		5,503 (15.3)	1,376 (16.5)		3,758 (10.5)	934 (11.2)	
10th-90th percentile	28,700 (80.0)	6,599 (79.3)		27,977 (78.0)	6,422 (77.2)		29,487 (82.2)	6,815 (81.9)	
>90th percentile	3,792 (10.6)	869 (10.4)		2,375 (6.6)	526 (6.3)		2,610 (7.3)	575 (6.9)	
FL			0.004			0.022			0.33
<10th percentile	3,227 (9.0)	842 (10.1)		3,776 (10.5)	963 (11.6)		1,113 (3.1)	277 (3.3)	
10th-90th percentile	30,402 (84.8)	6,948 (83.5)		28,641 (79.9)	6,579 (79.0)		32,728 (91.3)	7,556 (90.8)	
>90th percentile	2,226 (6.2)	534 (6.4)		3,438 (9.6)	782 (9.4)		2,014 (5.6)	491 (5.9)	
EFW			0.039			0.46			0.22
<10th percentile	2411 (6.7)	599 (7.2)		11,765 (32.8)	2,731 (32.8)		998 (2.8)	254 (3.1)	
10th-90th percentile	27369 (76.3)	6245 (75.0)		22,374 (62.4)	5,168 (62.1)		25,567 (71.3)	5,870 (70.5)	
>90th percentile	6075 (16.9)	1480 (17.8)		1,716 (4.8)	425 (5.1)		9,290 (25.9)	2,200 (26.4)	

* Presented as frequency, n (%), and compared using x^2 tests.

Table S2 Association between fetal biometry at 22-24 gestational week and subsequent GDM based on INTERGROWTH-21st standard.

Main exposure	GDM, n (%)	Crude OR (95% CI)	Adjusted OR† (95% CI)	Adjusted OR‡ (95% CI)
AC				
<10th percentile (n=2,329)	408 (17.5)	0.92(0.82,1.02)	0.98(0.87,1.09)	0.97(0.87,1.09)
10th-90th percentile (n=34,715)	6,538 (18.8)		1.0(reference)	
>90th percentile (n=7,135)	1,378 (19.3)	1.03(0.97,1.10)	0.97(0.91,1.04)	0.97(0.92,1.04)
HC				
<10th percentile (n=6,879)	1,376 (20.0)	1.09(1.02,1.16) *	1.15(1.08,1.23) *	1.12(1.05,1.20) *
10th-90th percentile (n=34,399)	6,422 (18.7)		1.0(reference)	
>90th percentile (n=2,901)	526 (18.1)	0.97(0.88,1.07)	0.92(0.84,1.02)	0.93(0.84,1.03)
FL				
<10th percentile (n=4,739)	963 (20.3)	1.11(1.03,1.20) *	1.17(1.08,1.26) *	1.14(1.05,1.23) *
10th-90th percentile (n=35,220)	6,579 (18.7)		1.0(reference)	
>90th percentile (n=4,220)	782 (18.3)	0.99(0.91,1.08)	0.95(0.88,1.04)	0.95(0.87,1.03)
EFW				
<10th percentile (n=14,496)	2,731 (18.8)	1.00(0.95,1.06)	1.06(1.01,1.12) *	1.05(1.00,1.11)
10th-90th percentile (n=27,542)	5,168 (18.8)		1.0(reference)	
>90th percentile (n=2,141)	425 (19.9)	1.08(0.96,1.20)	1.02(0.91,1.14)	1.00(0.89,1.12)

*P<0.05 †Adjusted for maternal age, parity and fetal sex. ‡Adjusted for maternal age, parity, fetal sex, pre-pregnancy height and pre-pregnancy BMI. OR=odds ratio; AC=abdominal circumference; HC=head circumference; FL=femur length; EFW=estimated fetal weight.

Table S3 Association between fetal biometry at 22-24 gestational weeks and subsequent GDM based on Chinese standard

Main exposure		GDM, n (%)	Crude OR (95% CI)	Adjusted OR† (95% CI)	Adjusted OR‡ (95% CI)
AC					
<10th percentile	(n=3,334)	594 (17.8)	0.93(0.85,1.02)	0.99(0.91,1.09)	1.00(0.90,1.09)
10th-90th percentile	(n=37,515)	7,078 (18.9)		1.0(reference)	
>90th percentile	(n=3,330)	653 (19.6)	1.05(0.96,1.14)	0.98(0.90,1.08)	0.97(0.88,1.06)
HC					
<10th percentile	(n=4,692)	934 (19.9)	1.07(0.99,1.16)	1.14(1.05,1.23) *	1.12(1.04,1.21) *
10th-90th percentile	(n=36,302)	6,811 (18.8)		1.0(reference)	
>90th percentile	(n=3,185)	575 (18.1)	0.91(0.83,1.01)	0.91(0.83,1.01)	0.92(0.83,1.01)
FL					
<10th percentile	(n=1,390)	277 (19.9)	1.07(0.94,1.22)	1.15(1.01,1.32) *	1.12(0.98,1.29)
10th-90th percentile	(n=40,284)	7,556 (18.8)		1.0(reference)	
>90th percentile	(n=2,505)	491 (19.6)	1.01(0.92,1.13)	1.01(0.92,1.13)	1.00(0.90,1.11)
EFW					
<10th percentile	(n=1,252)	254 (20.3)	1.11(0.96,1.27)	1.18(1.03,1.36) *	1.14(0.99,1.31)
10th-90th percentile	(n=31,437)	5,870 (18.7)		1.0(reference)	
>90th percentile	(n=11,490)	2,200 (19.1)	1.03(0.98,1.09)	0.97(0.92,1.03)	0.98(0.92,1.03)

*P<0.05 †Adjusted for maternal age, parity and fetal sex. ‡Adjusted for maternal age, parity, fetal sex, pre-pregnancy height and pre-pregnancy BMI. OR=odds ratio; AC=abdominal circumference; HC=head circumference; FL=femur length; EFW=estimated fetal weight.
